# Distinct subtypes of Alzheimer’s disease based on patterns of brain atrophy: longitudinal trajectories and clinical applications

**DOI:** 10.1038/srep46263

**Published:** 2017-04-18

**Authors:** Daniel Ferreira, Chloë Verhagen, Juan Andrés Hernández-Cabrera, Lena Cavallin, Chun-Jie Guo, Urban Ekman, J-Sebastian Muehlboeck, Andrew Simmons, José Barroso, Lars-Olof Wahlund, Eric Westman

**Affiliations:** 1 Department of Neurobiology, Care Sciences and Society, Centre for Alzheimer Research, Division of Clinical Geriatrics, Karolinska Institutet, Stockholm, Sweden; 2 Department of Psychology, Faculty of Social and Behavioural Sciences, Utrecht University, Utrecht, The Netherlands; 3 Faculty of Psychology, University of La Laguna, Tenerife, Spain; 4 Department of Clinical Science, Intervention and Technology, Division of Medical Imaging and Technology, Karolinska Institutet, Stockholm, Sweden; 5 Department of Radiology, Karolinska University Hospital in Huddinge, Huddinge, Sweden; 6 Department of Radiology, The First Hospital of Jilin University, Jilin, China; 7 Department of Neuroimaging, Centre for Neuroimaging Sciences, Institute of Psychiatry, Psychology and Neuroscience, King’s College London, London, UK; 8 NIHR Biomedical Research Centre for Mental Health, London, UK; 9 NIHR Biomedical Research Unit for Dementia, London, UK

## Abstract

Atrophy patterns on MRI can reliably predict three neuropathological subtypes of Alzheimer’s disease (AD): typical, limbic-predominant, or hippocampal-sparing. A method to enable their investigation in the clinical routine is still lacking. We aimed to (1) validate the combined use of visual rating scales for identification of AD subtypes; (2) characterise these subtypes at baseline and over two years; and (3) investigate how atrophy patterns and non-memory cognitive domains contribute to memory impairment. AD patients were classified as either typical AD (n = 100), limbic-predominant (n = 33), or hippocampal-sparing (n = 35) by using the Scheltens’ scale for medial temporal lobe atrophy (MTA), the Koedam’s scale for posterior atrophy (PA), and the Pasquier’s global cortical atrophy scale for frontal atrophy (GCA-F). A fourth group with no atrophy was also identified (n = 30). 230 healthy controls were also included. There was great overlap among subtypes in demographic, clinical, and cognitive variables. Memory performance was more dependent on non-memory cognitive functions in hippocampal-sparing and the no atrophy group. Hippocampal-sparing and the no atrophy group showed less aggressive disease progression. Visual rating scales can be used to identify distinct AD subtypes. Recognizing AD heterogeneity is important and visual rating scales may facilitate investigation of AD heterogeneity in clinical routine.

Alzheimer’s disease (AD) is a heterogeneous disease^1^,^2^,^3^,^4^,^5^. Current diagnostic criteria recognize this heterogeneity in the form of different cognitive presentations^6^,^7^,^8^. However, there is also neuropathological and structural heterogeneity^4^,^9^. Whitwell *et al*.^10^ grouped AD patients into amnestic and non-amnestic types. Amnestic patients evidenced atrophy in the medial temporal lobe, while non-amnestic patients showed atrophy in lateral regions of the parietal, temporal, and frontal lobes with relative sparing of the medial temporal lobes^10^. Subtyping based on the spread of neurofibrillary tangles (NFT) revealed fairly corresponding groups^4^. The amnestic form was highly represented on both the typical AD subtype (balanced NFT counts in the hippocampus and the associative cortex, i.e. lateral parietal, temporal, and frontal regions) and the limbic-predominant subtype (NFT counts predominantly in the hippocampus). The non-amnestic syndromes were more frequent in the atypical hippocampal-sparing AD subtype (NFT counts predominantly in the associative cortex). In a subsequent study, patterns of atrophy in MRI reliably tracked the distribution of NFT pathology at autopsy^9^. Hence, evidence suggests a connection between patterns of NFT spread, brain atrophy, and the cognitive presentation.

Recently, Byun *et al*.^11^ investigated these three subtypes as well as a fourth AD group with no atrophy by studying brain atrophy patterns on MRI data from the Alzheimer’s Disease Neuroimaging Initiative (ADNI-1^12^,^13^). Further, longitudinal progression over two years was studied. Limbic-predominant AD and the group with no atrophy showed slower progression than typical AD and hippocampal-sparing AD^11^. Data-driven approaches using MRI data have largely confirmed these pathologically defined subtypes^1^,^2^,^14^,^15^. Other authors have also applied data-driven approaches to cognitive data but the resulting subtypes differ noticeably from study to study^3^,^5^,^16^,^17^. However, data-driven approaches rely on group analysis and sophisticated methods that make them difficult to translate into clinical practice at present. Still, MRI is in a privileged position for studying AD heterogeneity because impairment in a given cognitive function may emerge from heterogeneous underlying neuropathology and atrophy patterns^8^,^9^,^10^,^18^.

We investigated whether visual rating scales of brain atrophy in MRI might be useful to capture the above-mentioned AD subtypes. Visual rating scales are quick and easy to use, and are the primary method for assessing brain structural changes in clinical settings^18^,^19^,^20^,^21^. However, visual rating scales are often used individually. Applying them in combination increases their diagnostic capacity and enables the study of patterns of brain atrophy^18^,^19^. We propose a way to easily identify patterns of atrophy using three visual rating scales covering the medial temporal, frontal and posterior cortices. We aimed to (1) validate the combined use of visual rating scales for identification of AD subtypes; (2) characterize the resulting subtypes at baseline and longitudinally over two years; and (3) since all the AD patients in our sample were amnestic, we also investigated how atrophy patterns and non-memory cognitive domains contribute to memory impairment, a relevant question not yet investigated in different AD subtypes. Thus, the three aims were addressed to facilitate investigation of the different AD subtypes in the clinical routine using already at-place and widely used clinical diagnostic tools.

## Results

### Clinical and cognitive characterization of the AD subtypes

**Figure 1:**
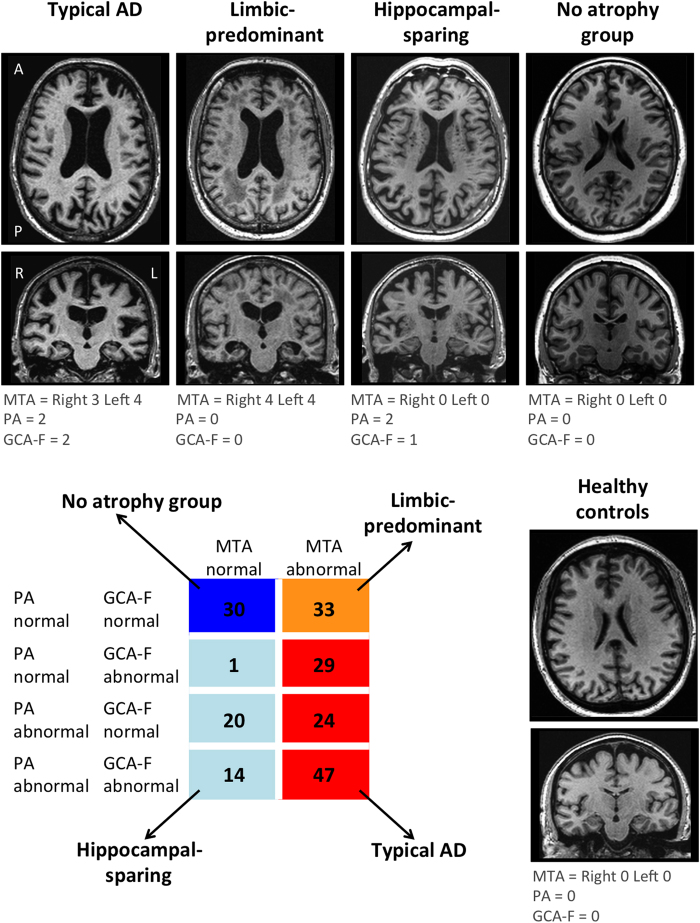
Subtypes of AD based on patterns of brain atrophy from visual rating scales. Regional atrophy was measured with the MTA, PA and GCA-F visual rating scales based only on T1-weigthed images. In the three visual rating scales, a score of zero denotes no atrophy, whereas scores from one to three (PA and GCA-F) or four (MTA) indicate an increasing degree of atrophy. The typical AD subtype was defined as abnormal MTA together with abnormal PA and/or abnormal GCA-F. The limbic-predominant subtype was defined as abnormal MTA alone with normal PA and GCA-F. The hippocampal-sparing subtype included abnormal PA and/or abnormal GCA-F, but normal MTA. The no atrophy group was defined as normal scores in MTA, PA, and GCA-F. The figure shows examples for each subtype as well as descriptive analysis on the different study groups. AD = Alzheimer’s disease; MTA = medial temporal atrophy scale; PA = posterior atrophy scale; GCA-F = global cortical atrophy scale – frontal subscale; A = anterior part of the brain; P = posterior part of the brain; R = right; L = left.

**Figure 2:**
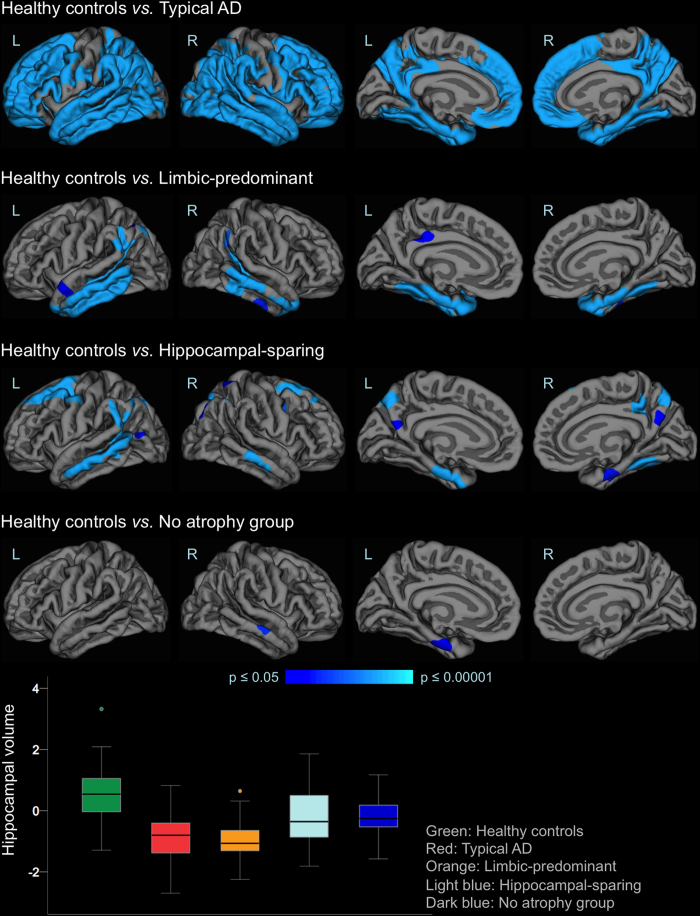
Cortical thickness and hippocampal volumes. The brain images show cortical maps of differences in thickness when comparing the different AD subtypes with the healthy controls. A general linear model was fitted at each vertex. Study group was entered as independent variable and TIV as a covariate. Z Monte Carlo simulations were conducted for cluster-forming with a threshold of p ≤ 0.001 (two-sided), yielding clusters corrected for multiple comparisons. Only vertexes belonging to clusters surviving this correction are displayed. Significant clusters were mapped on standard templates depicted in lateral (first two images on each row) and medial (last two images on each row) views, both for left (L) and right (R) hemispheres. The coloured bar illustrates the significance level of the differences (i.e. less cortical thickness in the AD patients) from dark blue (p ≤ 0.05) to light blue (p ≤ 0.00001). All these results stand after controlling for age, gender, years of education, and APOE ε4 status (data not shown). The boxplot represents the averaged hippocampal volume controlling for TIV, age, years of education, and APOE ε4 status. Hence, the y-axis represents adjusted and standardized values. Box values represent median and confidence intervals. The groups’ sizes are specified in Fig. 1 and Table 1. AD = Alzheimer’s disease.

Table 1 shows the main demographic and clinical characteristics of the study groups. Visual examples for each group are shown in Fig. 1. The largest group was typical AD (n = 100), as expected, present in 50.5% of the AD patients. The atypical subtypes were less prevalent and showed similar frequency: hippocampal-sparing (n = 35, 17.7%), limbic-predominant (n = 33, 16.7%), and no atrophy group (n = 30, 15.2%). Maps of cortical thickness as well as hippocampal volumes are displayed in Fig. 2.

**Figure 3:**
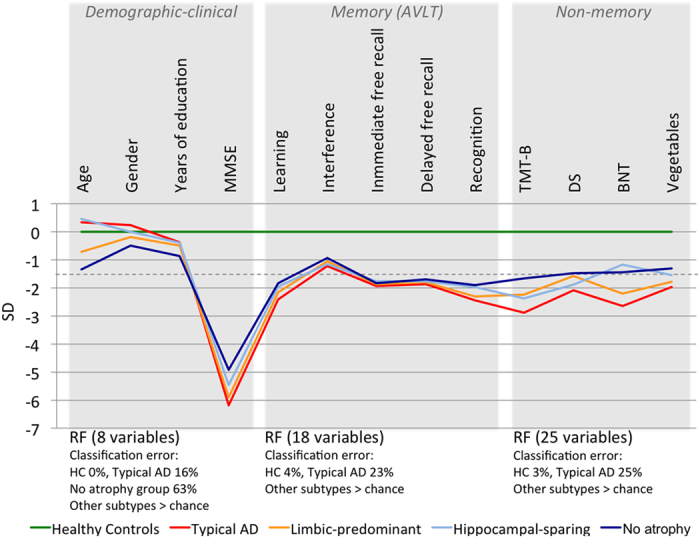
Demographic-clinical variables and cognitive profile. A selection of variables is reported for each random forest (RF) analysis. Classification error is reported only for the correctly classified study groups. The complete list of variables as well as full results from these models are detailed in Table 2. The dashed line shows the clinical cut-off of −1.5 standard deviations (SD). The random forest models reveal great overlap among subtypes with high comparability in demographic, clinical, and cognitive variables at baseline, including cerebrospinal fluid biomarkers, and APOE ε4 distribution. The groups’ sizes are specified in Fig. 1 and Table 1. AD = Alzheimer’s disease; HC = healthy controls; MMSE = mini-mental state examination; AVLT = Auditory Verbal Learning test; TMT-B = Trail Making Test part B; DS = digit symbol from the Wechsler Adult Intelligence Scale – Revised; BNT = Boston Naming Test.

Three random forest models were conducted to characterize the study groups according to (1) demographic-clinical variables, (2) memory variables, and (3) non-memory cognitive variables (see Table 2 for a list of variables included in each analysis as well as summary of results). Results showed great overlap (Fig. 3). Healthy controls and typical AD patients were correctly classified in the three models. Both resulted as the least and the most severe groups, respectively. However, any combination of the different sets of variables did not allow classifying limbic-predominant, hippocampal-sparing, and the no atrophy group better than chance. To note, the four AD subtypes were comparable on disease duration, CSF biomarkers and APOE ε4 distribution.

A follow-up mixed ANOVA was performed to further investigate the interaction between five memory components (within-subjects factor: total learning, interference, immediate free recall, delayed free recall, and recognition) and AD subtype (between-subjects factor). This interaction was statistically significant (F_(5, 324)_ = 3.419; p = 0.004). Impairment in the learning component was greater in typical AD than in both hippocampal-sparing (t_(86)_ = −3.427; p = 0.006) and the no atrophy group (t_(117)_ = −3.181; p = 0.010), but this effect was not observed for the other components (Fig. 3). These results hold after controlling for age, Mini-Mental state examination (MMSE), Clinical Dementia Rating (CDR), and disease duration (data not shown).

We then investigated whether these findings in memory could have any clinical relevance by classifying performance as normal or clinically impaired (−1.5 SD) using the healthy controls as reference group. Descriptive analyses confirmed that clinical impairment in learning was more frequent in typical AD than in the other subtypes (Table 3). Clinical impairment in delayed recall and recognition was more frequent in typical AD and limbic-predominant. The hippocampal-sparing group was the subtype having more interference effect. The gain variable (recognition minus delayed free recall) showed that hippocampal-sparing and specially the no atrophy group get more benefit from cues (recognition) than typical AD and limbic-predominant.

### Contribution of non-memory functions to memory performance

The results above suggest that despite memory impairment looks similar across AD subtypes, the nature of this memory impairment might be different, i.e. non-memory cognitive functions may be contributing differently to performance in memory across subtypes. To test for this we performed separate multiple linear regression models to investigate which non-memory components contributed the most to performance in memory across AD subtypes, and in comparison with the healthy controls. Due to the small sample size for some AD subtypes (n ≈ 30), results that were not stable in the non-parametric multiple regression model (i.e. dominance analysis) were rejected and not reported (Table 4). Only learning, delayed free recall and recognition where tested for simplicity.

In the healthy controls, learning was significantly associated with semantic abilities (β = 0.292) and processing speed (β = 0.229). The same pattern was obtained for typical AD and limbic-predominant, but for typical AD, lexical access was a significant predictor of learning as well (β = 0.219).

Delayed free recall was significantly predicted by learning alone in healthy controls (β = 0.744), typical AD (β = 0.560), and limbic-predominant (β = 0.483); and by both learning (β = 0.725) and attention/processing speed (β = −0.336) in the no atrophy group. In hippocampal-sparing, delayed free recall was associated to executive functioning (β = 0.548) and semantic abilities (β = 0.455), but not learning.

Finally, recognition was significantly predicted by learning alone in healthy controls (β = 0.469) and typical AD (β = 0.486); and by learning and lexical access in the no atrophy group (β = 0.445 and β = 0.357, respectively). Dominance analysis showed that semantic abilities are also important for recognition in the no atrophy group.

### Disease progression over two years

**Figure 4:**
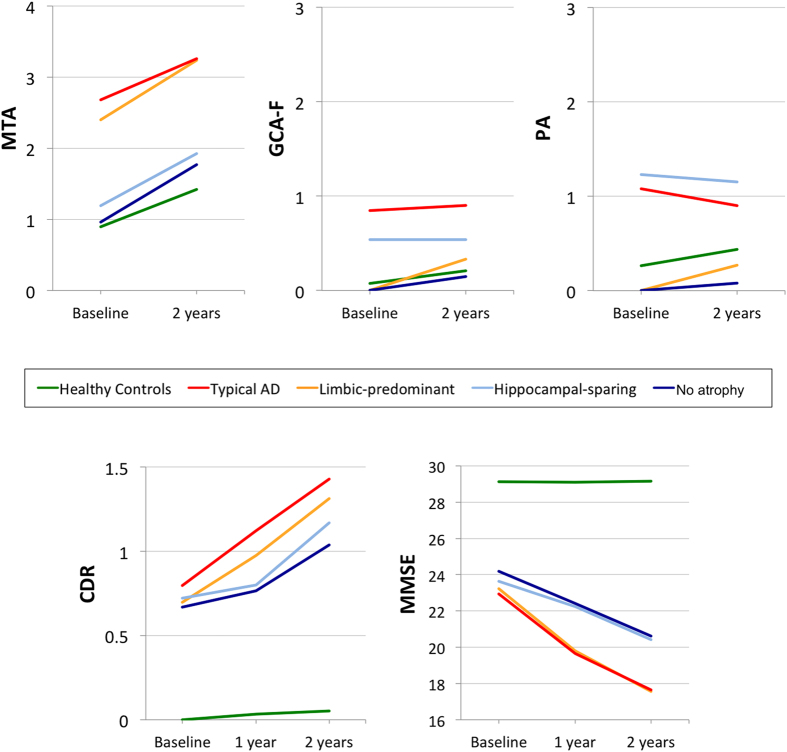
Disease progression over two years. Longitudinal scores for the visual rating scales (i.e. MTA, GCA-F, and PA) were available for 190 cases (110 healthy controls, 39 typical AD, 15 limbic-predominant, 13 hippocampal-sparing, 13 no atrophy group). Scores from MTA left and MTA right were averaged for simpler representation since longitudinal progression was similar in the two of them. Longitudinal values for CDR were available for 229 cases (147 healthy controls, 41 typical AD, 16 limbic-predominant, 12 hippocampal-sparing, 13 no atrophy group). Mixed effects analysis uses all data available at each time point. MTA = medial temporal atrophy visual rating scale; posterior atrophy visual rating scale; global cortical atrophy visual rating scale – frontal subscale; AD = Alzheimer’s disease; HC = healthy controls; CDR = clinical dementia rating; MMSE = mini-mental state examination.

Figure 4 shows the longitudinal progression in the visual rating scores across study groups.

The mixed effects model showed that clinical progression (CDR) over 2 years was faster in all the AD subtypes (estimates between 0.14 and 0.29) than in the healthy controls (estimate = 0.03) (F_(4, 690)_ = 46.896; p < 0.001) (Fig. 4). Moreover, the slope was greater in typical AD (estimate = 0.29) than in hippocampal-sparing (estimate = 0.16; t_(728)_ = −2.998; p = 0.015) and the no atrophy group (estimate = 0.14; t_(680)_ = −3.443; p < 0.001); and in limbic-predominant (estimate = 0.28) than in hippocampal-sparing (t_(708)_ = −2.374; trend for significance: p = 0.054) and the no atrophy group (t_(672)_ = −2.746; p = 0.024). No significant effects were obtained for the quadratic model, indicating that clinical progression in all the subtypes is lineal rather than quadratic.

Regarding global cognitive decline (MMSE), all the AD subtypes had faster decline over 2 years (estimates between −3.08 and −1.48) than the healthy controls (estimate = −0.01) (F_(4, 676)_ = 57.432; p < 0.001) (Fig. 4). Moreover, the slope was greater in typical AD (estimate = −2.69) than in hippocampal-sparing (estimate = −1.48; t_(704)_ = 3.152; p = 0.010); and in limbic-predominant (estimate = −3.08) than in hippocampal-sparing (t_(688)_ = 3.551; p < 0.001) and the no atrophy group (t_(663)_ = 2.850; p = 0.020). No significant effects were obtained for the quadratic model, indicating that cognitive decline in all the subtypes is lineal rather than quadratic. All these results stand after controlling for age, gender, and years of education (data not shown).

## Discussion

The AD subtypes investigated here have consistently been identified in previous studies. However, a method to enable their investigation in the clinical routine has been lacking so far. In the current study, visual rating scales were used as subtyping method because they are the primary method for assessing brain structural changes in clinical settings^18^,^19^,^20^,^21^. Although grouped data is reported, the visual rating scales can be applied at the individual level for clinical diagnosis. The aims in the current study were to (1) validate the combined use of visual rating scales for identification of AD subtypes; (2) characterize the resulting subtypes; and (3) investigate how atrophy patterns and non-memory cognitive domains contribute to memory impairment. The findings show that visual rating scales help to identify distinct AD subtypes with different disease progression. However, routine cognitive and clinical evaluations, CSF biomarkers and APOE ε4 genotype did not allow such discrimination, which may limit their clinical use for subtypes identification. Typical AD was the most impaired subtype at baseline and together with limbic predominant AD had worse clinical progression. Identification of patients with hippocampal-sparing and no atrophy is also clinically relevant since they showed less aggressive disease progression.

Visual rating scales are feasible and reproducible^19^,^21^,^22^,^23^,^24^,^25^. Intra-rater values (weighted κ) are usually around 0.90 for MTA^19^,^23^, and between 0.70 and 0.90 for GCA and PA^19^,^23^,^25^. Inter-rater values (weighted κ) are usually around 0.85 for MTA^23^, and between 0.60 and 0.80 for GCA and PA^23^,^25^. These weighted κ values correspond to substantial and almost perfect agreement^26^, thus proving their reproducibility.

The subtypes in this study were defined a-priori based on converging evidence suggesting three AD subtypes^1^,^2^,^3^,^4^,^5^,^9^,^10^,^11^,^14^,^15^. Vertex analyses in the current study showed that visual rating scales can successfully identify patterns of atrophy similar to those depicted by previous sophisticated MRI studies^1^,^2^,^11^,^14^,^15^, and coherent with those tracking the spread of NFT^9^. The prevalence of different subtypes obtained in this study are very similar to those previously reported, where typical AD usually includes 50–75% of the AD patients, limbic-predominant is referred in around 15–35%, and hippocampal-sparing in around 10–25%^1^,^4^,^9^,^11^,^15^. In the only study identifying a no atrophy group, the prevalence was 10%^11^. Since visual rating scales can be easily applied in the clinical routine^18^, this finding may have significant impact for current diagnosis and management of AD patients in clinical settings. The fact that the four subtypes were rather comparable on cognition, CSF biomarkers and APOE ε4 distribution at baseline further supports the use of MRI to identify clinically relevant subtypes. Different longitudinal progression of these subtypes supports this approach as well.

There was great overlap in the clinical and cognitive profiles, especially among the atypical AD subtypes, which highlights the difficulty in detecting these in routine clinical evaluations by only using clinical or cognitive measurements. Despite this, when comparing our results with other studies, the characteristics of our AD subtypes are largely comparable with what has previously been reported. The typical AD subtype has previously been found to be among the oldest^1^,^2^,^4^,^9^,^11^,^15^, have later onset^1^,^4^,^9^,^11^,^15^, include a higher frequency of males^2^,^11^, and have similar disease duration to limbic-predominant and hippocampal-sparing^1^,^9^,^11^. Contrary to our finding, hippocampal-predominant AD has been found to be among the oldest groups^1^,^4^,^9^,^15^ and to have shorter disease duration^14^. An explanation could be that atrophy in the medial temporal lobe is frequent in normal aging, hence clinical cut-offs for the medial temporal atrophy (MTA) scale are age-corrected^19^. This could lead to a younger hippocampal-predominant subtype in the present study. Age-corrections were not performed in previous studies except in Byun *et al*.^11^ and Varol *et al*.^2^, who indeed showed consistent results with our findings. Also contrary to our finding, hippocampal-sparing has been related with younger age^1^,^4^,^9^,^15^, earlier onset^1^,^4^,^9^,^14^,^15^, and shorter disease duration^4^. These results in previous studies could be explained by higher prevalence of early-onset AD in their samples, known to display predominant posterior atrophy and a more aggressive presentation^9^,^27^. However, early-onset AD is not common in ADNI-1, our sample, where posterior atrophy possibly results from a different process perhaps related or amplified by increased age.

To our knowledge, only our study and the one by Byun *et al*.^11^ have investigated a group of AD patients with no atrophy. Although data from ADNI-1 was used in both studies, we included 198 AD patients while Byun *et al*.^11^ included 163 AD patients. Most of the drops in Byun *et al*.^11^ corresponds to the hippocampal-sparing and the no atrophy group groups. Visual inspection of the demographic and clinical characteristics reveals that both groups are largely comparable, although our no atrophy group is younger and has higher frequency of females. An age correction was performed in both studies but the gender correction was not performed in our study because scores in our scales of posterior and frontal atrophy are not influenced by gender, and scores in MTA are only marginaly influenced by gender^19^. Thus, the gender correction and the fact that most of the droped cases in the no atrophy group from Byun *et al*.^11^ were young females, could be the explanation for these differences.

Interactions between memory components and contribution of non-memory cognitive functions were investigated for the first time in a study of this kind. Learning capacity was compromised in the four subtypes, but typical AD and limbic-predominant evidenced more consolidation problems, while hippocampal-sparing and the no atrophy group showed more problems in free retrieval of information. In addition, hippocampal-sparing was the group showing greater vulnerability to interference. Similar results were obtained in another study using ADNI-1 data^5^. The cognitive profiles discussed above were further confirmed in the regression/dominance analysis, where delayed recall and recognition were more dependent on non-memory cognitive functions in hippocampal-sparing and the no atrophy group than in typical AD and limbic-predominant. These profiles are coherent with the underlying pattern of brain atrophy. Noh *et al*.^1^ found prominent memory impairment in their parietal predominant AD subtype (analogous to hippocampal-sparing). The authors suggested that memory deficits might be associated with attention and working memory dysfunction in their parietal predominant subtype^1^. Hence, despite great overlap among AD subtypes, the nature of memory impairment seems to be different^5^. In this regard, Whitwell *et al*.^9^ made an important observation: a patient with Alzheimer’s dementia dominated by memory impairment can have any of the three subtypes of AD. This is exactly what we demonstrate in the current study, since all our AD patients have memory impairment, and we further demonstrate that the determinants of this memory impairment are varied and correspond with different atrophy patterns. This finding may have clinical utility, both for diagnosis/prognosis and cognitive interventions. Likewise, it is very important to note that these cognitive profiles were obtained using multivariate statistical methods on grouped data. Such information can be very difficult to find at the individual level in clinical routine, not to mention with often used cognitive tests. Thus, the use of visual rating scales seems to be extremely beneficial in this context.

One limitation in the current study is that ADNI-1 is a quite homogeneous sample. All AD patients fulfil the amnestic criteria at entry and aspects such as vascular pathology are excluded. Our results should thus be replicated in a more heterogeneous clinical sample that also includes non-amnestic AD presentations. It could also be argued that the different subtypes identified here reflect AD patients at different stages of the disease (e.g. typical AD being a later stage of limbic-predominant AD), rather than truly distinct AD subtypes. However, it has been demonstrated that these subtypes result from differential spread of NFT^4^. Further, no differences were observed on scales that stage the disease such as CDR, neither on disease duration. Related to this, it could be argued that the no atrophy group might be an initial stage of the disease but, again, no differences on CDR or disease duration were obtained. Alternatively, it could be argued that visual rating scales might not be sensitive enough to capture subtle atrophy in this no atrophy group. However, automated MRI methods confirmed this lack of atrophy in our vertex analysis, and a similar result was obtained using voxel-based morphometry in another ADNI-1 study^11^. Finally, we performed group analyses in order to characterize the different AD subtypes. Due to large within-group variability, there was great overlapping between subtypes and some conclusions especially on the non-imaging variables may be difficult to translate to the individual level, where clinical diagnosis takes place.

In conclusion, visual rating scales can be used to identify distinct and clinically relevant AD subtypes. To note, these subtypes could not be properly discriminated based on other common clinical tools such as cognitive tests, CSF biomarkers or APOE ε4 distribution. It has previously been argued that heterogeneity in AD complicates overcoming the two main challenges in the field at present, namely, discovery of disease-modifying treatments and achievement of accurate diagnosis and clinical prognosis^1^,^2^,^3^,^10^,^28^. It has also been discussed that advancing in the knowledge on different AD subtypes could shed some light to recently failed clinical trials by enabling tailored treatments in more homogeneous subgroups of patients^2^,^28^. The same would also help to better discriminate between highly overlapping clinical phenotypes such as AD with significant frontal involvement and frontotemporal lobe dementia^4^,^10^,^18^,^29^. Findings from the current study suggest that visual rating scales may facilitate investigation of AD heterogeneity in clinical routine. Implementing AD subtypes based on visual rating scales in the clinical routine should be easy and it is warranted to do as soon as possible in order to test its potential clinical impact. Whether using this method clinically may improve diagnosis and prognosis in “real world” AD patients stills needs to be determined.

## Methods

### Participants

AD patients and healthy controls from the ADNI-1 (adni.loni.usc.edu, PI Michael M. Weiner) with longitudinal data available at 1 and 2 years follow-up were selected for the current study, giving a total of 198 AD patients and 230 healthy controls. ADNI was launched in 2003 by the National Institute on Aging, the National Institute of Biomedical Imaging and Bioengineering, the Food and Drug Administration, private pharmaceutical companies, and non-profit organizations^12^. The project was established to develop standardized imaging techniques and biomarkers in AD research. The AD patients and healthy controls were clinically diagnosed following standard procedures as detailed before^19^. Of note, memory impairment was required for all the AD patients based on Logical Memory II (Wechsler Memory Scale – Revised, WMS-R^30^). All diagnoses were made without the use of MRI scans. The study was approved by the institutional review boards of all participating ADNI centres. Written informed consent was obtained from all participants or authorized representatives after extensive description of the ADNI according to the Declaration of Helsinki. All methods were performed in accordance with the relevant guidelines and regulations.

### Magnetic resonance imaging, automated image processing, and visual rating scales

A 3D T1-weighted magnetization-prepared rapid gradient-echo (MPRAGE) sequence was acquired on 1.5 T MRI scanners (voxel size 1.1 × 1.1 × 1.2 mm^3^)^12^. TheHiveDB Database system^31^ was used to automatically process the images with FreeSurfer 5.3.0, following previously described standard procedures^32^. This procedure provides measurements of cortical thickness at the vertex level, as well as a measurement of the total intracranial volume (TIV).

Regional atrophy was measured with visual rating scales based only the T1-weigthed images as detailed elsewhere^19^. Briefly, atrophy in the medial temporal lobe was evaluated with the MTA scale^33^; 2) atrophy in the posterior cortex was evaluated with the posterior atrophy (PA) scale^23^; and 3) atrophy in the frontal lobe was evaluated with the global cortical atrophy scale – frontal subscale (GCA-F)^25^. The MTA scale scores the degree of atrophy from zero to four in the hippocampus, parahippocampal gyrus, entorhinal cortex and the surrounding cerebrospinal fluid spaces. The PA scale scores the degree of atrophy from zero to three in the posterior cingulate sulcus, precuneus, parieto-occipital sulcus and the parietal cortex. The GCA-F scale scores the degree of atrophy from zero to three in the frontal lobe as delimited by the central sulcus, the frontal bone and the fissure of Sylvius. Therefore, the three scales primarily reflect cortical atrophy. Nonetheless, these scales also indirectly reflect ventricular enlargement since the inferior lateral ventricles are considered in MTA ratings, and GCA and PA ratings are based on widening of sulci, which is frequently correlated with ventricular enlargement. This is relevant because AD patients have more brain atrophy and larger ventricles than controls in the ADNI-1 cohort^34^. On the other hand, these scales are not designed to capture white matter hyperintensities (WMH), which are commonly regarded as markers of cerebrovascular disease. However, WMH burden in ADNI-1 is minimal relative to other cohorts^35^ due to exclusion of individuals with a Hachinski Ischemic Score^36^ of more than or equal to 5.

In the three visual rating scales, a score of zero denotes no atrophy, whereas scores from one to four indicate an increasing degree of atrophy. MTA analysis was based on coronal reconstructions, GCA-F on axial reconstructions and PA on reconstructions from all three planes. The images were rated both at baseline and at two years follow-up. Subtyping is based on baseline ratings, while longitudinal ratings were used to study disease progression over two years.

All cases were rated by an experienced radiologist (L.C.). Intra-rater reliability in 120 random cases achieved a weighted κ of 0.94 and 0.89 for MTA in left and right hemispheres, respectively, 0.88 for PA, and 0.83 for GCA-F. The same 120 random cases were also rated by a newly trained radiologist (C.-J.G.) for inter-rater analysis: weighted κ of 0.71 and 0.70 for MTA in left and right hemispheres, respectively, 0.88 for PA, and 0.79 for GCA-F. Both raters were blind to any information about the participants.

### AD subtypes based on patterns of brain atrophy

Deviation from normality was established following a recently proposed list of practical cut-offs^19^. The MTA scores ≥1.5, ≥1.5, ≥2, ≥2.5 were considered abnormal for the respective age ranges 45–64, 65–74, 75–84, and 85–94 years. A previous study using the same dataset as here demonstrated that an age-correction does not improve PA and GCA-F diagnostic performance^19^. Therefore, the same cut-off was used for PA and GCA-F. A score ≥1 was considered abnormal irrespectively of the age range^19^. The three AD subtypes identified in previous literature^4^,^9^ were defined based on the combination of MTA, PA, and GCA-F as follows (see also Fig. 1). The typical AD subtype was defined as atrophy in the medial temporal lobe (abnormal MTA) together with atrophy in the posterior cortex (abnormal PA) and/or frontal cortex (abnormal GCA-F). The limbic-predominant subtype was defined as atrophy in the medial temporal lobe alone (abnormal MTA with normal PA and GCA-F). The hippocampal-sparing subtype included atrophy in the posterior cortex (abnormal PA) and/or frontal cortex (abnormal GCA-F), but not in the medial temporal lobe (normal MTA). A group with no atrophy was also identified as in Byun *et al*.^11^ when AD patients displayed normal scores in MTA, PA, and GCA-F.

### Demographic and clinical variables

Age, gender, and years of education were included as demographic variables. Clinical severity was assessed with the CDR^37^ scale and global cognition with the MMSE^38^. FAQ^39^ was used to measure functional activities of daily living and GDS^40^ to measure depressive symptomatology. Age at disease onset, disease duration, and APOE ε4 status were also measured. Memory was assessed with the Auditory Verbal Learning test (AVLT)^41^. The different AVLT items were used to investigate different memory components. In particular, the sum of the five learning trials of the list A reflects learning capacity. Performance in the list B served as an estimate of interference effects during learning. Interference effects are frequently interpreted as distortions of existing memories possibly due to source-monitoring deficits. Free recall of the list A right after recall of the list B measures immediate recall. Free recall of the list A 30 minutes after learning measures delayed recall. Recognition reflects the ability to identify previously learned items from the list A. Finally, gain was calculated by subtracting the recognition percentage from the delayed percentage in order to quantify benefit from additional help when retrieving stored information. Executive functions (Trail Making Test part B, TMT-B)^42^, attention/processing speed (Digit Symbol, DS, from the Wechsler Adult Intelligence Scale – Revised, WAIS-R)^43^, language (Boston Naming Test, BNT)^44^, and semantic abilities (semantic fluency, vegetables)^45^ were also assessed. Digit span from the WMS-R^30^ and the Clock Test^46^ were further included for the random forest models (see statistical analysis). CSF samples were available for 102 AD patients and 115 healthy controls. Complete procedure descriptions are available at www.adni-info.org.

### Statistical analysis

Mixed effects models (fixed and random effects) and mixed ANOVA/ANCOVA (split plot) were used to analyse the interaction between a between-subjects factor (study group) and a within-subjects factor (memory component and time). In the mixed effects models, the fixed-effect factors were study group, time, and the study group-by-time interaction. The random effect factor was the participants. When time was included in the model, both linear and quadratic effects were tested in order to investigate whether for example disease progression is linear or gets accelerated/decelerated after a certain time point. Multiple linear regression (backwards) was performed to analyse the contribution of non-memory cognitive functions to different memory components. Confirmatory dominance analyses, an extension of multiple regression, were performed with a non-parametric test based on bootstrapping (1000 iterations), which is less vulnerable to small sample sizes. Random forest analysis (500 trees) was also used to investigate differences between groups in multiple variables while avoiding multiple testing. P-values in all principal and post-hoc analyses were adjusted with the Benjamini-Hochberg’s^47^ correction for multiple comparisons. Model assumptions were tested in all the cases by visual inspection of residuals and data distribution, as well as by inspecting the pertinent statistical parameters. Results were considered significant when p ≤ 0.05 (two-tailed).

Image analyses based on the vertex across the cortical mantle were carried out using FreeSurfer software as detailed elsewhere^48^. Briefly, maps were smoothed using a circularly symmetric Gaussian kernel across the surface with a full width at half maximum (FWHM) of 10 mm. A general linear model was fitted at each vertex. Study group was entered as independent variable and TIV as a covariate. Z Monte Carlo simulations were used with a cluster-forming threshold of p ≤ 0.001 (two-sided), yielding results corrected for multiple comparisons.

## Additional Information

**How to cite this article:** Ferreira, D. *et al*. Distinct subtypes of Alzheimer’s disease based on patterns of brain atrophy: longitudinal trajectories and clinical applications. *Sci. Rep.* **7**, 46263; doi: 10.1038/srep46263 (2017).

**Publisher's note:** Springer Nature remains neutral with regard to jurisdictional claims in published maps and institutional affiliations.

## Supplementary Material

Supplementary Material

## Figures and Tables

**Table 1:** Characteristics of the AD subtypes and healthy controls.

	Healthy controls (n = 230)	Alzheimer’s disease subtypes				
		Typical AD (n = 100, 50.5%)	Limbic-predominant (n = 33, 16.7%)	Hippocampal-sparing (n = 35, 17.7%)	No atrophy group (n = 30, 15.2%)	p-value
Age	75.9 (5.0)	77.7 (6.4)	72.3 (7.1)^a,b^	78.2 (8.2)^c^	69.2 (6.9)^a,b,d^	<0.001
Gender, % female	48.7	37.0	57.6	48.6	73.3^b^	0.016
Years of education	16.0 (2.9)	15.0 (3.4)^a^	14.6 (2.2)	14.9 (3.1)	13.6 (3.7)^a^	<0.001
MMSE	29.1 (1.0)	22.9 (2.1)^a^	23.2 (1.9)^a^	23.7 (2.2)^a^	24.2 (1.5)^a,b^	<0.001
CDR total	0 (0)	0.8 (0.2)^a^	0.7 (0.2)^a,b^	0.7 (0.3)^a^	0.7 (0.2)^a,b^	<0.001
Age at onset^e^	—	73.6 (7.2)	68.6 (6.9)^b^	76.3 (7.6)^c^	66.8 (7.1)^b,d^	<0.001
Disease duration^e^	—	3.8 (2.6)	3.3 (2.2)	3.2 (2.7)	2.6 (1.4)	0.189
ApoE status, % ε4 allele	26.5	69.0^a^	69.7^a^	51.4^a^	73.3^a^	<0.001
CSF Aß_1-42_, % abnormal^f^	40.0	96.0^a^	90.0^a^	85.0^a^	91.7^a^	<0.001
CSF T-tau, % abnormal^g^	21.2	56.0^a^	75.0^a^	75.0^a^	66.7^a^	<0.001

The table shows mean (SD) except for Gender, APOE status and the CSF biomarkers, where percentage is reported. ^a^Significantly different from Healthy controls; ^b^Significantly different from Typical AD; ^c^Significantly different from Limbic-predominant; ^d^Significantly different from Hippocampal-sparing. ^e^N = 154; ^f^N = 217; ^g^N = 215. AD = Alzheimer’s disease; CDR = clinical dementia rating; MMSE = mini-mental state examination; APOE = apolipoprotein E; CSF = cerebrospinal fluid; Aß_1-42_ = amyloid-ß-peptide 1–42; T-tau = total level of tau protein.

**Table 2:** Random Forest models.

(A) Description of variables included in the models		
Model 1	Model 2	Model 3
Demographic-clinical variables	Memory variables	Non-memory cognitive variables
Age	AVLT Learning trial 1	WAIS-R Digit Symbol
Gender	AVLT Learning trial 2	WMS-R Digit forward span
Years of education	AVLT Learning trial 3	WMS-R Digit forward length
APOE status	AVLT Learning trial 4	WMS-R Digit backward span
GDS	AVLT Learning trial 5	WMS-R Digit backward length
FAQ	AVLT Learning trial 1 errors	TMT-A correct answers
CDR	AVLT Learning trial 2 errors	TMT-A commission errors
MMSE	AVLT Learning trial 3 errors	TMT-A omission errors
CSF Aß_42_^a^	AVLT Learning trial 4 errors	TMT-B correct answers
CSF Total tau^a^	AVLT Learning trial 5 errors	TMT-B commission errors
CSF p-tau^a^	AVLT B (interference)	TMT-B omission errors
Disease duration^a^	AVLT B (interference) errors	Verbal Fluency – vegetables
Age of onset^a^	AVLT immediate free recall	Verbal Fluency – vegetables intrusions
	AVLT immediate free recall errors	Verbal Fluency – vegetables perseverations
	AVLT delayed free recall	Verbal Fluency – animals
	AVLT delayed free recall errors	Verbal Fluency – animals intrusions
	AVLT recognition	Verbal Fluency – animals perseverations
	AVLT recognition errors	BNT total
		BNT spontaneous
		BNT number semantic cues
		BNT spontaneous + semantic
		BNT number phonetic cues
		BNT spontaneous + semantic + phonetic
		Clock Test drawing
		Clock Test copy
**(B) Summary of results**		
**Model 1**	**Model 2**	**Model 3**
N = 415	N = 408	N = 398
Error by chance = 80%	Error by chance = 80%	Error by chance = 80%
Classification error:	Classification error:	Classification error:
Healthy controls: 0%	Healthy controls: 4%	Healthy controls: 3%
Typical AD: 16%	Typical AD: 23%	Typical AD: 25%
Limbic-predominant: 88%	Limbic-predominant: 100%	Limbic-predominant: 100%
Hippocampal-sparing: 82%	Hippocampal-sparing: 100%	Hippocampal-sparing: 97%
No atrophy group: 63%	No atrophy group: 100%	No atrophy group: 100%

^a^The same model was repeated including CSF biomarkers in all the sample (model 1’), and CSF biomarkers + disease duration + age of onset only in Alzheimer’s disease patients (model 1”). Results obtained were the same as in model 1. Results presented in the manuscript Fig. 3 are those from model 1 (8 variables). APOE = apolipoprotein E; GDS = geriatric depression scale; FAQ = functional activities questionnaire; CDR = clinical dementia rating; MMSE = mini-mental state examination; CSF = cerebrospinal fluid; Aß_1-42_ = amyloid-ß-peptide 1–42; T-tau = total level of tau protein; AVLT = Auditory Verbal Learning test; WAIS-R = Wechsler Adult Intelligence Scale – Revised; WMS-R = Wechsler Memory Scale – Revised; TMT-B = Trail Making Test part B; BNT = Boston Naming Test; AD = Alzheimer’s disease.

**Table 3:** Percentage of AD patients with clinical impairment (−1.5 SD) across memory components.

	Typical AD (n = 100)	Limbic-predominant (n = 33)	Hippocampal-sparing (n = 35)	No atrophy group (n = 30)
Learning^a^	88.0	75.8	81.8	74.1
Interference^b^	36.6	33.3	42.6	29.6
Immediate^c^	78.0	66.7	63.6	66.7
Delayed^d^	87.1	87.9	82.4	74.1
Recognition^d^	74.2	78.8	64.7	51.9
Gain^d^	12.9	9.1	17.7	22.2

Values in the table represent percentage. The healthy control group was used as reference for calculating the clinical cut-off of −1.5 SD. Interference was based on the AVLT list B item in order to estimate interference effects during learning. A higher percentage of individuals showing clinical impairment (−1.5 SD) in this variable reflects greater interference effect, which is frequently interpreted as distortions of existing memories possibly due to source-monitoring deficits. Gain was calculated by subtracting the recognition percentage from the delayed percentage in order to quantify benefit from additional help when retrieving stored information: higher gain values represent more retrieving problems. ^a^N = 411; ^b^N = 412; ^c^N = 408; ^d^N = 414. AD = Alzheimer’s disease.

**Table 4:** Contribution of non-memory cognitive functions to different memory measurements.

		TMT-B	DS	BNT	Vegetables	Learning
**Learning**						
	**Multiple regression model**					
Healthy controls	F_(2, 222)_ = 22.065; p < 0.001; R^2^adj. = 16%		**0.229**		**0.292**	—
Typical AD	F_(3, 80)_ = 15.880; p < 0.001; R^2^adj. = 35%		**0.264**	**0.219**	**0.363**	—
Limbic-predominant	F_(2, 30)_ = 13.526; p < 0.001; R^2^adj. = 44%		**0.340**		**0.510**	—
Hippocampal-sparing	*non significant*					—
No atrophy group	*non significant*					—
	**Dominance analysis (bootstrapping)**					
Healthy controls	Explained variance = 18%	1%	**6%**	1%	**10%**	—
Typical AD	Explained variance = 38%	4%	**8%**	**9%**	**17%**	—
Limbic-predominant	Explained variance = 49%	10%	**11%**	5%	**23%**	—
**Delayed free recall**						
	**Multiple regression model**					
Healthy controls	F_(1, 223)_ = 276.979; p < 0.001; R^2^adj. = 55%					**0.744**
Typical AD	F_(2, 81)_ = 15.080; p < 0.001; R^2^adj. = 25%		−0.249*			**0.560**
Limbic-predominant	F_(1, 31)_ = 9.435; p = 0.008; R^2^adj. = 21%					**0.483**
Hippocampal-sparing	F_(2, 27)_ = 5.225; p = 0.012; R^2^adj. = 23%	**0.548**			**0.455**	
No atrophy group	F_(2, 22)_ = 17.815; p < 0.001; R^2^adj. = 58%		—**0.336**			**0.725**
	**Dominance analysis (bootstrapping)**					
Healthy controls	Explained variance = 56%	0%	3%	1%	3%	**49%**
Typical AD	Explained variance = 28%	0%	2%*	1%	2%	**23%**
Limbic-predominant	Explained variance = 26%	1%	2%	0%	2%	**21%**
Hippocampal-sparing	Explained variance = 33%	**14%**	5%	3%	**10%**	1%
No atrophy group	Explained variance = 66%	6%	**8%**	2%	6%	**44%**
**Recognition**						
	**Multiple regression model**					
Healthy controls	F_(1, 223)_ = 62.858; p < 0.001; R^2^adj. = 22%					**0.469**
Typical AD	F_(2, 81)_ = 11.366; p < 0.001; R^2^adj. = 20%	0.253*				**0.486**
Limbic-predominant	*non significant*					
Hippocampal-sparing	*non significant*					
No atrophy group	F_(2, 22)_ = 5.890; p = 0.027; R^2^adj. = 29%			**0.357**		**0.445**
	**Dominance analysis (bootstrapping)**					
Healthy controls	Explained variance = 24%	1%	1%	1%	1%	**20%**
Typical AD	Explained variance = 24%	3%*	1%	2%	1%	**17%**
No atrophy group	Explained variance = 37%	1%	1%	**8%**	10%	**17%**

Values in the table represent standardized beta (for multiple regression model) and the fraction of the variance explained by a predictor (dominance analysis). In addition to multiple regression analyses, confirmatory dominance analyses were performed due to the small sample size in some of the AD subtypes. The asterisk (*) in the table indicates when a significant predictor in the regression models (in bold) was rejected due to lack of stability in the dominance analysis (i.e. the percentage in bold is low, or equal to the one in not-bold). All p-values are corrected with Hochberg’s correction for multiple comparisons. AD = Alzheimer’s disease; adj. = adjusted; TMT-B = Trail Making Test part B; DS = digit symbol from the Wechsler Adult Intelligence Scale – Revised; BNT = Boston Naming Test.
