# Supplementary material for: Distinct subtypes of Alzheimer’s disease based on patterns of brain atrophy: longitudinal trajectories and clinical applications
Source: Sci Rep. 2017 Apr 18;7:46263. doi: 10.1038/srep46263 (PMC5394684; doi:10.1038/srep46263)
Supplement: Supplementary Material [file srep46263-s1.pdf]

Distinct subtypes of Alzheimer's disease based on patterns of brain atrophy: longitudinal trajectories and clinical applications

Daniel Ferreira, PhD; Chloë Verhagen, MSc; Juan Andrés Hernández-Cabrera, PhD; Lena Cavallin, MD, PhD; Chun-Jie Guo, MD, PhD; Urban Ekman, PhD; J-Sebastian Muehlboeck, MSc; Andrew Simmons, PhD; José Barroso, PhD; Lars-Olof Wahlund, MD, PhD; and Eric Westman, PhD

SUPPLEMENTARY MATERIAL

Supplementary Table 1. Contributors from the ADNI consortium.

Michael W. Weiner, MD (UC San Francisco, Principal Investigator, Executive Committee, Initial Concept Planning & Development, Early Project Proposal Development); Paul Aisen, MD (UC San Diego, ADCS PI and Director of Coordinating Center Clinical Core and Executive Committee); Ronald Petersen, MD, PhD (Mayo Clinic, Executive Committee); Clifford R. Jack, Jr., MD (Mayo Clinic, Executive Committee); William Jagust, MD (UC Berkeley, Executive Committee); John Q. Trojanowki, MD, PhD (U Pennsylvania, Executive Committee); Arthur W. Toga, PhD (USC, Executive Committee); Laurel Beckett, PhD (UC Davis, Executive Committee); Robert C. Green, MD, MPH (Brigham and Women's Hospital/Harvard Medical School, Executive Committee, Data and Publication Committee); John Morris, MD (Washington University St. Louis, Executive Committee); Leslie M. Shaw (University of Pennsylvania, Executive Committee); Zaven Khachaturian, PhD (Prevent Alzheimer's Disease 2020, ADNI External Advisory Board, Initial Concept Planning & Development, Early Project Proposal Development); Greg Sorensen, MD (Siemens, ADNI External Advisory Board); Maria Carrillo, PhD (Alzheimer's Association, ADNI External Advisory Board); Lew Kuller, MD (University of

Pittsburg, ADNI External Advisory Board); Marc Raichle, MD (Washington University St. Louis, ADNI External Advisory Board); Steven Paul, MD (Cornell University, ADNI External Advisory Board, Early Project Proposal Development); Peter Davies, MD (Albert Einstein College of Medicine of Yeshiva University, ADNI External Advisory Board); Howard Fillit, MD (AD Drug Discovery Foundation, ADNI External Advisory Board); Franz Hefti, PhD (Acumen Pharmaceuticals, ADNI External Advisory Board); Davie Holtzman, MD (Washington University St. Louis, ADNI External Advisory Board); Marcel M. Mesulam, MD (Northwestern University, ADNI External Advisory Board); William Potter, MD (National Institute of Mental Health, ADNI External Advisory Board, Early Project Proposal Development); Peter Snyder, PhD (Brown University, ADNI External Advisory Board, Early Project Proposal Development); Adam Schwartz, MD (Eli Lilly, ADNI 2 Private Partner Scientific Board); Tom Montine, MD, PhD (University of Washington, Resource Allocation Review Committee); Ronald Petersen, MD, PhD (Mayo Clinic, Clinical Core Leader); Paul Aisen, MD (UC San Diego, Clinical Core Leader); Ronald G. Thomas, PhD (UC San Diego, Clinical Informatics and Operations); Michael Donohue, PhD (UC San Diego, Clinical Informatics and Operations); Sarah Walter, MSc (UC San Diego, Clinical Informatics and Operations); Devon Gessert (UC San Diego , Clinical Informatics and Operations); Tamie Sather, MA (UC San Diego, Clinical Informatics and Operations); Gus Jiminez, MBS (UC San Diego , Clinical Informatics and Operations); Archana B. Balasubramanian, PhD (UC San Diego, Clinical Informatics and Operations); Jennifer Mason, MPH (UC San Diego, Clinical Informatics and Operations); Iris Sim (UC San Diego, Clinical Informatics and Operations); Laurel Beckett, PhD (UC Davis, Biostatistics Core Leader); Danielle Harvey, PhD (UC Davis, Biostatistics Core Key Personnel); Michael Donohue, PhD (UC San Diego, Biostatistics Core Leader); Clifford R. Jack, Jr., MD (Mayo Clinic, MRI Core Leader); Matthew Bernstein, PhD (Mayo Clinic, MRI Core Key Personnel); Nick Fox, MD (University of London, MRI Core Key Personnel); Paul Thompson, PhD (UCLA School of Medicine, MRI Core Key Personnel); Norbert Schuff, PhD (UCSF MRI, MRI Core Key Personnel); Charles DeCArli, MD (UC Davis, MRI Core Key Personnel); Bret Borowski, RT (Mayo Clinic, MRI Core Key Personnel); Jeff Gunter, PhD (Mayo Clinic, MRI Core Key Personnel); Matt Senjem, MS (Mayo Clinic, MRI Core Key

Personnel); Prashanthi Vemuri, PhD (Mayo Clinic , MRI Core Key Personnel); David Jones, MD (Mayo Clinic, MRI Core Key Personnel); Kejal Kantarci (Mayo Clinic, MRI Core Key Personnel); Chad Ward (Mayo Clinic, MRI Core Key Personnel); William Jagust, MD (UC Berkeley, PET Core Leader); Robert A. Koeppe, PhD (University of Michigan, PET Core Key Personnel); Norm Foster, MD (University of Utah, PET Core Key Personnel); Eric M. Reiman, MD (Banner Alzheimer's Institute, PET Core Key Personnel); Kewei Chen, PhD (Banner Alzheimer's Institute, PET Core Key Personnel); Chet Mathis, MD (University of Pittsburgh, PET Core Key Personnel); Susan Landau, PhD (UC Berkeley, PET Core Key Personnel); John C. Morris, MD (Washington University St. Louis, Neuropathology Core Leader); Nigel J. Cairns, PhD, MRCPath (Washington University St. Louis, , Neuropathology Core Leader); Erin Householder (Washington University St. Louis, Neuropathology Core Leader); Lisa Taylor-Reinwald, BA, HTL (Washington University St. Louis, Neuropathology Core Leader); Leslie M. Shaw, PhD (UPenn School of Medicine, Biomarkers Core Leader); John Q. Trojanowki, MD, PhD (UPenn School of Medicine, Biomarkers Core Leader); Virginia Lee, PhD, MBA (UPenn School of Medicine, Biomarkers Core Key Personnel); Magdalena Korecka, PhD (UPenn School of Medicine, Biomarkers Core Key Personnel); Michal Figurski, PhD (UPenn School of Medicine, Biomarkers Core Key Personnel); Arthur W. Toga, PhD (USC, Informatics Core Leader); Karen Crawford (USC, Informatics Core Key Personnel); Scott Neu, PhD (USC, Informatics Core Key Personnel); Andrew J. Saykin, PsyD (Indiana University, Genetics Core Leader); Tatiana M. Foroud, PhD (Indiana University, Genetics Core Key Personnel); Steven Potkin, MD (UC Irvine, Genetics Core Key Personnel); Li Shen, PhD (Indiana University, Genetics Core Key Personnel); Kelley Faber, MS, CCRC (Indiana University, Genetics Core Key Personnel); Sungeun Kim, PhD (Indiana University, Genetics Core Key Personnel); Kwangsik Nho, PhD (Indiana University, Genetics Core Key Personnel); Lean Thal, MD (UC San Diego, Initial Concept Planning & Development, Early Project Proposal Development); Neil Buckholtz National (Insititute on Aging, Early Project Proposal Development); Marylyn Albert, PhD (Johns Hopkins University, Early Project Proposal Development); Richard Frank, MD, PhD (Richard Frank Consulting, Early Project Proposal Development); John Hsiao, MD (National Institute on Aging); Jeffrey Kaye, MD (Oregon Health and Science University, site investigator); Joseph

Quinn, MD (Oregon Health and Science University, site investigator); Lisa Silbert, MD (Oregon Health and Science University, site investigator); Betty Lind, BS (Oregon Health and Science University, site investigator); Raina Carter, BA Sara Dolen, BS (Oregon Health and Science University, Past Investigator); Lon S. Schneider, MD (University of Southern California, site investigator); Sonia Pawluczyk, MD (University of Southern California, site investigator); Mauricio Beccera, BS (University of Southern California, site investigator); Liberty Teodoro, RN (University of Southern California, site investigator); Bryan M. Spann, DO, PhD (University of Southern California, Past Investigator); James Brewer, MD, PhD (University of California--San Diego, site investigator); Helen Vanderswag, RN (University of California--San Diego, site investigator); Adam Fleisher, MD (University of California--San Diego, Past Investigator); Judith L. Heidebrink, MD, MS (University of Michigan, Site Investigator); Joanne L. Lord, LPN, BA, CCRC (University of Michigan, Site Investigator); Ronald Petersen, MD, PhD (Mayo Clinic, Site Investigator); Sara S. Mason, RN (Mayo Clinic, Site Investigator); Colleen S. Albers, RN (Mayo Clinic, Site Investigator); David Knopman, MD (Mayo Clinic, Site Investigator); Kris Johnson, RN (Mayo Clinic, Past Investigator); Rachelle S. Doody, MD, PhD (Baylor College of Medicine, Site Investigator); Javier Villanueva-Meyer, MD (Baylor College of Medicine, Site Investigator); Munir Chowdhury, MBBS, MS (Baylor College of Medicine, Site Investigator); Susan Rountree, MD (Baylor College of Medicine, Site Investigator); Mimi Dang, MD (Baylor College of Medicine, Site Investigator); Yaakov Stern, PhD (Columbia University Medical Center, Site Investigator); Lawrence S. Honig, MD, PhD (Columbia University Medical Center, Site Investigator); Karen L. Bell, MD (Columbia University Medical Center, Site Investigator); Beau Ances, MD (Washington University St. Louis, Site Investigator); John C. Morris, MD (Washington University St. Louis, Site Investigator); Maria Carroll, RN, MSN (Washington University St. Louis, Site Investigator); Mary L. Creech, RN, MSW (Washington University St. Louis, Site Investigator); Erin Franklin, MS, CCRP (Washington University St. Louis, Site Investigator); Mark A. Mintun, MD (Washington University St. Louis, Past Investigator); Stacy Schneider, APRN, BC, GNP (Washington University St. Louis, Past Investigator); Angela Oliver, RN, BSN, MSG (Washington University St. Louis, Past Investigator); Daniel Marson, JD, PhD (University of Alabama – Birmingham,

Site Investigator); Randall Griffith, PhD, ABPP (University of Alabama – Birmingham, Site Investigator); David Clark, MD (University of Alabama – Birmingham, Site Investigator); David Geldmacher, MD (University of Alabama – Birmingham, Site Investigator); John Brockington, MD (University of Alabama – Birmingham, Site Investigator); Erik Roberson, MD (University of Alabama – Birmingham, Site Investigator); Marissa Natelson Love, MD (University of Alabama – Birmingham, Site Investigator); Hillel Grossman, MD (Mount Sinai School of Medicine, Site Investigator); Effie Mitsis, PhD (Mount Sinai School of Medicine, Site Investigator); Raj C. Shah, MD (Rush University Medical Center, Site Investigator); Leyla deToledo-Morrell, PhD (Rush University Medical Center, Past Investigator); Ranjan Duara, MD (Wien Center, Site Investigator); Daniel Varon, MD (Wien Center, Site Investigator); Maria T. Greig, MD (Wien Center, Site Investigator); Peggy Roberts, CNA (Wien Center, Past Investigator); Marilyn Albert, PhD (Johns Hopkins University, Site Investigator); Chiadi Onyike, MD (Johns Hopkins University, Site Investigator); Daniel D'Agostino II, BS (Johns Hopkins University, Site Investigator); Stephanie Kielb, BS (Johns Hopkins University, Site Investigator, Past Investigator); James E. Galvin, MD, MPH (New York University, Site Investigator); Brittany Cerbone (New York University, Site Investigator); Christina A. Michel (New York University, Past Investigator); Dana M. Pogorelec (New York University, Past Investigator); Henry Rusinek, PhD (New York University, Past Investigator); Mony J de Leon, EdD (New York University, Past Investigator); Lidia Glodzik, MD, PhD (New York University, Past Investigator); Susan De Santi, PhD (New York University, Past Investigator); Murali P. Doraiswamy, MBBS, FRCP (Duke University Medical Center, Site Investigator); Jeffrey R. Petrella, MD (Duke University Medical Center, Site Investigator); Salvador Borges-Neto, MD (Duke University Medical Center, Site Investigator); Terence Z. Wong, MD (Duke University Medical Center, Past Investigator); Edward Coleman (Duke University Medical Center, Past Investigator); Steven E. Arnold, MD (University of Pennsylvania, Site Investigator); Jason H. Karlawish, MD (University of Pennsylvania, Site Investigator); David Wolk, MD (University of Pennsylvania, Site Investigator); Christopher M. Clark, MD (University of Pennsylvania, Site Investigator); Charles D. Smith, MD (University of Kentucky, Site Investigator); Greg Jicha, MD (University of Kentucky, Site Investigator); Peter Hardy, PhD

(University of Kentucky, Site Investigator); Partha Sinha, PhD (University of Kentucky, Site Investigator); Elizabeth Oates, MD (University of Kentucky, Site Investigator); Gary Conrad, MD (University of Kentucky, Site Investigator); Oscar L. Lopez, MD (University of Pittsburgh, Site Investigator); MaryAnn Oakley, MA (University of Pittsburgh, Site Investigator); Donna M. Simpson, CRNP, MPH (University of Pittsburgh, Site Investigator); Anton P. Porsteinsson, MD (University of Rochester Medical Center, Site Investigator); Bonnie S. Goldstein, MS, NP (University of Rochester Medical Center, Site Investigator); Kim Martin, RN (University of Rochester Medical Center, Site Investigator); Kelly M. Makino, BS (University of Rochester Medical Center, Past Investigator); M. Saleem Ismail, MD (University of Rochester Medical Center, Past Investigator); Connie Brand, RN (University of Rochester Medical Center, Past Investigator); Ruth A. Mulnard, DNSc, RN, FAAN (University of California, Irvine, Site Investigator); Gaby Thai, MD (University of California, Irvine, Site Investigator); Catherine Mc-Adams-Ortiz, MSN, RN, A/GNP (University of California, Irvine, Site Investigator); Kyle Womack, MD (University of Texas Southwestern Medical School, Site Investigator); Dana Mathews, MD, PhD (University of Texas Southwestern Medical School, Site Investigator); Mary Quiceno, MD (University of Texas Southwestern Medical School, Site Investigator); Allan I. Levey, MD, PhD (Emory University, Site Investigator); James J. Lah, MD, PhD (Emory University, Site Investigator); Janet S. Cellar, DNP, PMHCNS-BC (Emory University, Site Investigator); Jeffrey M. Burns, MD (University of Kansas, Medical Center, Site Investigator); Russell H. Swerdlow, MD (University of Kansas, Medical Center, Site Investigator); William M. Brooks, PhD (University of Kansas, Medical Center, Site Investigator); Liana Apostolova, MD (UCLA, Site Investigator); Kathleen Tingus, PhD (UCLA, Site Investigator); Ellen Woo, PhD (UCLA, Site Investigator); Daniel H.S. Silverman, MD, PhD (UCLA, Site Investigator); Po H. Lu, PsyD (UCLA, Past Investigator); George Bartzokis, MD (UCLA, Past Investigator); Neill R Graff-Radford, MBBCH, FRCP (London) (Mayo Clinic, Jacksonville, Site Investigator); Francine Parfitt, MSH, CCRC (Mayo Clinic, Jacksonville, Site Investigator); Tracy Kendall, BA, CCRP (Mayo Clinic, Jacksonville, Site Investigator); Heather Johnson, MLS, CCRP (Mayo Clinic, Jacksonville, Past Investigator); Martin R. Farlow, MD (Indiana University, Site Investigator); Ann Marie Hake, MD (Indiana University, Site Investigator);

Brandy R. Matthews, MD (Indiana University, Site Investigator); Jared R. Brosch, MD (Indiana University, Site Investigator); Scott Herring, RN, CCRC (Indiana University, Past Investigator); Cynthia Hunt, BS, CCRP (Indiana University, Past Investigator); Christopher H. van Dyck, MD (Yale University School of Medicine, Site Investigator); Richard E. Carson, PhD (Yale University School of Medicine, Site Investigator); Martha G. MacAvoy, PhD (Yale University School of Medicine, Site Investigator); Pradeep Varma, MD (Yale University School of Medicine, Site Investigator); Howard Chertkow, MD (McGill Univ., Montreal-Jewish General Hospital, Site Investigator); Howard Bergman, MD (McGill Univ., Montreal-Jewish General Hospital, Site Investigator); Chris Hosein, MEd (McGill Univ., Montreal-Jewish General Hospital, Site Investigator); Sandra Black, MD, FRCPC (Sunnybrook Health Sciences, Ontario, Site Investigator); Bojana Stefanovic, PhD (Sunnybrook Health Sciences, Ontario, Site Investigator); Curtis Caldwell, PhD (Sunnybrook Health Sciences, Ontario, Site Investigator); Ging-Yuek Robin Hsiung, MD, MHSc, FRCPC (U.B.C. Clinic for AD & Related Disorders, Site Investigator); Howard Feldman, MD, FRCPC (U.B.C. Clinic for AD & Related Disorders, Site Investigator); Benita Mudge, BS (U.B.C. Clinic for AD & Related Disorders, Site Investigator); Michele Assaly, MA (U.B.C. Clinic for AD & Related Disorders, Past Investigator); Elizabeth Finger, MD (Cognitive Neurology - St. Joseph's, Ontario, Site Investigator); Stephen Pasternack, MD, PhD (Cognitive Neurology - St. Joseph's, Ontario, Site Investigator); Irina Rachisky, MD (Cognitive Neurology - St. Joseph's, Ontario, Site Investigator); Dick Trost, PhD (Cognitive Neurology - St. Joseph's, Ontario, Past Investigator); Andrew Kertesz, MD (Cognitive Neurology - St. Joseph's, Ontario, Past Investigator); Charles Bernick, MD, MPH (Cleveland Clinic Lou Ruvo Center for Brain Health, Site Investigator); Donna Munic, PhD (Cleveland Clinic Lou Ruvo Center for Brain Health, Site Investigator); Marek-Marsel Mesulam, MD (Northwestern University, Site Investigator); Kristine Lipowski, MA (Northwestern University, Site Investigator); Sandra Weintraub, PhD (Northwestern University, Site Investigator); Borna Bonakdarpour, MD (Northwestern University, Site Investigator); Diana Kerwin, MD (Northwestern University, Past Investigator); Chuang-Kuo Wu, MD, PhD (Northwestern University, Past Investigator); Nancy Johnson, PhD(Northwestern University, Past Investigator); Carl Sadowsky, MD (Premiere Research Inst (Palm Beach Neurology), Site Investigator); Teresa Villena,

MD (Premiere Research Inst (Palm Beach Neurology), Site Investigator); Raymond Scott Turner, MD, PhD (Georgetown University Medical Center, Site Investigator); Kathleen Johnson, NP (Georgetown University Medical Center, Site Investigator); Brigid Reynolds, NP (Georgetown University Medical Center, Site Investigator); Reisa A. Sperling, MD (Brigham and Women's Hospital, Site Investigator); Keith A. Johnson, MD (Brigham and Women's Hospital, Site Investigator); Gad Marshall, MD (Brigham and Women's Hospital, Site Investigator); Jerome Yesavage, MD (Stanford University, Site Investigator); Joy L. Taylor, PhD (Stanford University, Site Investigator); Barton Lane, MD (Stanford University, Site Investigator); Allyson Rosen, PhD (Stanford University, Past Investigator); Jared Tinklenberg, MD (Stanford University, Past Investigator); Marwan N. Sabbagh, MD (Banner Sun Health Research Institute, Site Investigator); Christine M. Belden, PsyD (Banner Sun Health Research Institute, Site Investigator); Sandra A. Jacobson, MD (Banner Sun Health Research Institute, Site Investigator); Sherye A. Sirrel, CCRC (Banner Sun Health Research Institute, Site Investigator); Neil Kowall, MD (Boston University, Site Investigator); Ronald Killiany, PhD (Boston University, Site Investigator); Andrew E. Budson, MD (Boston University, Site Investigator); Alexander Norbash, MD (Boston University, Past Investigator); Patricia Lynn Johnson, BA (Boston University, Past Investigator); Thomas O. Obisesan, MD, MPH (Howard University, Site Investigator); Saba Wolday, MSc (Howard University, Site Investigator); Joanne Allard, PhD (Howard University, Site Investigator); Alan Lerner, MD (Case Western Reserve University, Site Investigator); Paula Ogrocki, PhD (Case Western Reserve University, Site Investigator); Curtis Tatsuoka, PhD (Case Western Reserve University, Site Investigator); Parianne Fatica, BA, CCRC (Case Western Reserve University, Site Investigator); Evan Fletcher, PhD (University of California, Davis – Sacramento, Site Investigator); Pauline Maillard, PhD (University of California, Davis – Sacramento, Site Investigator); John Olichney, MD (University of California, Davis – Sacramento, Site Investigator); Charles DeCarli, MD (University of California, Davis – Sacramento, Past Investigator); Owen Carmichael, PhD (University of California, Davis – Sacramento, Past Investigator); Smita Kittur, MD (Neurological Care of CNY, Past Investigator); Michael Borrie, MB ChB (Parkwood Hospital, Site Investigator); T-Y Lee, PhD (Parkwood Hospital, Site Investigator); Rob Bartha, PhD (Parkwood Hospital, Site Investigator); Sterling Johnson, PhD

(University of Wisconsin, Site Investigator); Sanjay Asthana, MD (University of Wisconsin, Site Investigator); Cynthia M. Carlsson, MD, MS (University of Wisconsin, Site Investigator); Steven G. Potkin, MD (University of California, Irvine - BIC, Site Investigator); Adrian Preda, MD(University of California, Irvine - BIC, Site Investigator); Dana Nguyen, PhD (University of California, Irvine - BIC, Site Investigator); Pierre Tariot, MD (Banner Alzheimer's Institute, Site Investigator); Anna Burke, MD (Banner Alzheimer's Institute, Site Investigator); Nadira Trncic, MD, PhD, CCRC (Banner Alzheimer's Institute, Site Investigator); Adam Fleisher, MD (Banner Alzheimer's Institute, Past Investigator); Stephanie Reeder, BA (Banner Alzheimer's Institute, Site Investigator); Vernice Bates, MD (Dent Neurologic Institute, Site Investigator); Horacio Capote, MD (Dent Neurologic Institute, Site Investigator); Michelle Rainka, PharmD, CCRP (Dent Neurologic Institute, Site Investigator); Douglas W. Scharre, MD (Ohio State University, Site Investigator); Maria Kataki, MD, PhD (Ohio State University, Site Investigator); Anahita Adeli, MD (Ohio State University, Site Investigator); Earl A. Zimmerman, MD (Albany Medical College, Site Investigator); Dzintra Celmins, MD (Albany Medical College, Site Investigator); Alice D. Brown, FNP (Albany Medical College, Site Investigator); Godfrey D. Pearlson, MD (Hartford Hospital, Olin Neuropsychiatry Research Center, Site Investigator); Karen Blank, MD (Hartford Hospital, Olin Neuropsychiatry Research Center, Site Investigator); Karen Anderson, RN (Hartford Hospital, Olin Neuropsychiatry Research Center, Site Investigator); Laura A. Flashman, PhD (Dartmouth-Hitchcock Medical Center, Site Investigator); Marc Seltzer, MD (Dartmouth-Hitchcock Medical Center, Site Investigator); Mary L. Hynes, RN, MPH (Dartmouth-Hitchcock Medical Center, Site Investigator); Robert B. Santulli, MD (Dartmouth-Hitchcock Medical Center, Past Investigator); Kaycee M. Sink, MD, MAS (Wake Forest University Health Sciences, Site Investigator); Leslie Gordineer (Wake Forest University Health Sciences, Site Investigator); Jeff D. Williamson, MD, MHS (Wake Forest University Health Sciences, Past Investigator); Pradeep Garg, PhD (Wake Forest University Health Sciences, Past Investigator); Franklin Watkins, MD (Wake Forest University Health Sciences, Past Investigator); Brian R. Ott, MD (Rhode Island Hospital, Site Investigator); Henry Querfurth, MD (Rhode Island Hospital, Site Investigator); Geoffrey Tremont, PhD (Rhode Island Hospital, Site Investigator); Stephen Salloway, MD, MS (Butler Hospital, Site Investigator); Paul

Malloy, PhD (Butler Hospital, Site Investigator); Stephen Correia, PhD (Butler Hospital, Site Investigator); Howard J. Rosen, MD (UC San Francisco, Site Investigator); Bruce L. Miller, MD (UC San Francisco, Site Investigator); David Perry, MD (UC San Francisco, Site Investigator); Jacobo Mintzer, MD, MBA (Medical University South Carolina, Site Investigator); Kenneth Spicer, MD, PhD (Medical University South Carolina, Site Investigator); David Bachman, MD (Medical University South Carolina, Site Investigator); Elizabether Finger, MD (St. Joseph's Health Care, Site Investigator); Stephen Pasternak, MD (St. Joseph's Health Care, Site Investigator); Irina Rachinsky, MD (St. Joseph's Health Care, Site Investigator); John Rogers, MD (St. Joseph's Health Care, Site Investigator); Andrew Kertesz, MD (St. Joseph's Health Care, Past Investigator); Dick Drost, MD (St. Joseph's Health Care, Past Investigator); Nunzio Pomara, MD (Nathan Kline Institute, Site Investigator); Raymundo Hernando, MD (Nathan Kline Institute, Site Investigator); Antero Sarrael, MD (Nathan Kline Institute, Site Investigator); Susan K. Schultz, MD (University of Iowa College of Medicine, Site Investigator); Laura L. Boles Ponto, PhD (University of Iowa College of Medicine, Site Investigator); Hyungsub Shim, MD (University of Iowa College of Medicine, Site Investigator); Karen Ekstam Smith, RN (University of Iowa College of Medicine, Site Investigator); Norman Relkin, MD, PhD (Cornell University, Site Investigator); Gloria Chaing, MD (Cornell University, Site Investigator); Michael Lin, MD (Cornell University, Site Investigator); Lisa Ravdin, PhD (Cornell University, Site Investigator); Amanda Smith, MD (University of South Florida: USF Health Byrd Alzheimer's Institute, Site Investigator); Balebail Ashok Raj, MD (University of South Florida: USF Health Byrd Alzheimer's Institute, Site Investigator); Kristin Fargher, MD (University of South Florida: USF Health Byrd Alzheimer's Institute, Past Investigator).
